# Designing for flourishing: a conceptual model for enhancing older adults’ well-being with social robots

**DOI:** 10.3389/frobt.2025.1607373

**Published:** 2025-08-20

**Authors:** Chantal Klier, Birgit Lugrin

**Affiliations:** Chair of Computer Science V (Socially Interactive Agents), University of Würzburg, Würzburg, Germany

**Keywords:** social robots, PERMA, self-determination theory, basic psychological needs, elderly, seniors, application design, measurement

## Abstract

This article gives a new perspective on designing robotic applications in elderly care with a special focus on socially assistive robots and seniors’ well-being. While various applications have been proposed there is currently no common conceptual model in designing interventions with social robots for seniors. Therefore, we propose a conceptual model that identifies five key domains for designing applications for socially interactive robots to enhance seniors’ well-being. We base our conceptual model on established theories from the social sciences. Namely, we propose that application design should consider integrating Self-Determination Theory by addressing the three basic psychological needs (autonomy, competence, and relatedness) to enhance seniors’ wellbeing. Furthermore, we recommend assessing the impact of social robots on well-being using the five building blocks of the PERMA framework: positive emotions, engagement, relationships, meaning, and accomplishment. By integrating these theoretical perspectives, researchers and developers gain a structured approach to designing social robot applications for cognitively healthy older adults and evaluating their effects.

## 1 Introduction

The global population is aging, and in Germany alone, a shortage of 690,000 caregivers is projected by 2049 (Statistisches Bundesamt, 2024). Even where staff are available, many residents in nursing homes remain unoccupied for extended periods (Ice, 2002). Consequently, the integration of robotic technologies into elderly care has been the focus of research for several years with various types of assistive robots serving different purposes. While numerous prototypes and research platforms exist, such as robotic drink assistants (Schroer et al., 2015) or walking aid robots (Zhao et al., 2022), these devices typically do not prioritize social interaction, are often expensive or not for sale.

In contrast, this paper focuses on socially interactive robots (Naseer et al., 2025) that are both affordable and commercially available. Often referred to as companion robots (Broekens et al., 2009), these robots are primarily designed to enhance the wellbeing of older adults (Bemelmans et al., 2012). Interventions using the seal robot Paro have demonstrated promising effects regarding individuals with dementia, such as improved mood (Wada et al., 2002), reduced loneliness (Robinson et al., 2013), and lower blood pressure (Robinson et al., 2015). Other notable applications include Pepper playing personalized music to stimulate memory in dementia patients (Kok et al., 2018), and NAO being used in therapy to reduce apathy (Valentí et al., 2015). Yet, a review of randomized controlled trials by Pu et al. (Pu et al., 2019) found no significant impact on the quality of life of older adults, most of whom had cognitive impairments. Still, their narrative review suggested improvements in engagement and interaction.

For seniors before the onset of dementia, often with mild cognitive impairments, promising approaches using social robots for cognitive training have been explored (Carolis et al., 2020), showing improvements in both cognition and emotional health (Vogan et al., 2020). Interestingly, Góngora Alonso et al. (Góngora Alonso et al., 2019) reported that elderly individuals without dementia or only mild cognitive impairments are less likely to accept robots, as they often do not see a need for them and wish to avoid being stigmatized as dependent (Wu et al., 2014). This reluctance may be related to the passive nature of robots like Paro, which have frequently been used in wellbeing studies in recent years (Scoglio et al., 2019). In contrast, more interactive robots offering quizzes or sing-alongs tend to help healthy older adults avoid boredom and develop a sense of attachment to the robot (Lee and Shin, 2024).

While much of the existing research centers on individuals with dementia or cognitive impairments (Abdi et al., 2018), there is a growing recognition of the need to explore how cognitively healthy older adults are affected by social robots (Nichol et al., 2024). When examining this group, there are already promising findings. For instance, Papadopoulos et al. (Papadopoulos et al., 2022) reported significant improvements in emotional wellbeing after 2 weeks of interaction with Pepper. Similarly, using NAO as a fitness coach led to high levels of likeability among seniors (Antonioni et al., 2021). Moreover, suggestions from social robots, whether physical, cognitive, or social, are perceived as persuasive (Hammer et al., 2016).

Building on this potential, the present paper focuses specifically on cognitively healthy older adults. Additionally, we do not adopt a deficit-oriented approach, such as aiming to reduce loneliness (Gasteiger et al., 2021a) or targeting depression (Yen et al., 2024). Instead, our focus is on fostering meaningful, enjoyable interactions that are emotionally rewarding, thereby promoting wellbeing and potentially preventing the emergence of negative emotional states.

Currently, much of the wellbeing oriented research emphasizes behavior change, such as promoting healthy eating (Robinson et al., 2020), or implementing positive psychology coaching (Jeong et al., 2020). However, we argue that a broader range of applications, beyond those aimed at behavior change, can contribute to maintaining or enhancing elderly wellbeing, provided they are intentionally designed with this goal in mind.

Therefore, in this paper, we propose a conceptual model grounded in psychological theory to guide the design of robotic applications for cognitively healthy older adults. In line with the need for theory-driven approaches (Trainum et al., 2023), our model combines the five building blocks of wellbeing from Seligman’s PERMA framework (Seligman, 2011) with core principles from Self-Determination Theory (SDT), specifically the fulfillment of basic psychological needs. While the integration of SDT into social robot design has also been suggested by Janssen and Schadenberg (Janssen and Schadenberg, 2024), through the METUX model (Peters et al., 2018) their approach focuses on user satisfaction and the development of new robots.

In contrast, our model is tailored to support the development of empirically testable applications for existing social robots in real-world settings. By grounding the design process in well-established theories and employing validated measures for both wellbeing and psychological need satisfaction, our model also addresses the call for a standardized approach to evaluate the effectiveness of social robots in elderly care (Andtfolk et al., 2022).

## 2 Theoretical foundation

In this section, we outline the theoretical foundation of our conceptual model. We begin by introducing Self-Determination Theory and its current relevance in the context of social robots. Next, we present three well-established models of wellbeing and discuss which we consider most suitable for our conceptual model.

### 2.1 Self-Determination Theory

SDT, proposed by Deci and Ryan (Deci and Ryan, 1985), comprises several so-called *mini-theories*. One of these is the Basic Psychological Needs Theory, which states that three fundamental needs must be satisfied to foster intrinsic motivation, personal growth, and wellbeing (Deci and Ryan, 2000). These universal needs are (Ryan et al., 2002):

• *Autonomy*: experiencing one’s actions as self-endorsed and aligned with personal interests and values, rather than being externally controlled.
• *Competence*: driving to pursue challenges that match one’s abilities, fostering continuous improvement and confidence in one’s actions.
• *Relatedness*: feeling emotionally connected to others, experiencing mutual care, and having a sense of belonging.

Research indicates that nursing home residents whose psychological needs are met report higher levels of wellbeing (Ferrand et al., 2014). Furthermore, greater perceived autonomy is associated with increased satisfaction (Bangerter et al., 2017). Interestingly, residents seem to prioritize relatedness over autonomy and competence, though with considerable individual variation (Custers et al., 2012). Notably, an SDT-driven physical activity intervention showed greater improvements in depression scores compared to a control group (Liu et al., 2024) and structured activities in long-term care reduce ill-being, primarily through enhanced relatedness (Duncan et al., 2017).

While the integration of those needs into social robotics has been limited, some studies showed promising results. Within the context of learning, van Minkelen et al. (van Minkelen et al., 2020) demonstrated that an SDT-supportive robot lead to increased task engagement and motivation in children. Recently, a social robot designed according to SDT principles significantly increased adult learners’ motivation compared to a control robot without such design (Lu et al., 2023). Given that SDT is a well-established framework for learning (Willems et al., 2012), it is not surprising that current integrations of SDT in social robotics predominantly focus on learning contexts.

In contrast, SDT-driven design has received little attention in social robotics for elderly care. We argue that this approach is particularly well-suited to the domain, as social robots naturally support psychological needs through their interactive presence. Unlike traditional care routines with fixed schedules, robots can offer on-demand activities, promoting autonomy and complementing occupational therapists (Wilson et al., 2016). They can also adapt content to individual skill levels, fostering competence more effectively than group activities led by a human caregiver. While they cannot replace human emotional bonds, their embodied and multimodal interactions (Breazeal et al., 2016) offer a more socially engaging experience than screen-based tools like tablets (Deublein et al., 2020), thereby supporting relatedness.

Several studies have indeed shown that the presence of social robots can support wellbeing among cognitively non-impaired older adults by offering companionship (Ge and Schleimer, 2023), emotional support (Tan et al., 2024), or being a game partner (Khosla et al., 2013). A promising example integrating SDT is the robotic exercise coach by Fasola and Mataric (Fasola and Mataric, 2013), which fostered intrinsic motivation by supporting older users’ autonomy. Based on these findings, we suggest that deliberately incorporating SDT into design could enhance the effectiveness of such interventions in promoting psychological well-being.

Beyond emphasizing the integration of SDT, we argue that the design of robotic applications should also be grounded in a coherent and appropriate framework of psychological wellbeing. Therefore, in the following section, we discuss which model we consider most suitable for guiding and assessing the impact of an SDT-oriented design on the wellbeing of older adults.

### 2.2 Psychological wellbeing

Psychological wellbeing is often set equal with “happiness”, aligning with a hedonic perspective (Kahneman, 1999). Psychological hedonism states that human behavior is primarily driven by wanting to either experience pleasure or avoid pain (Moore et al., 2019). In contrast, the eudaimonic perspective suggests that the basis of wellbeing is leading a meaningful life, marked by personal growth and self-realization (Waterman, 1993). Over the past decades, psychologists have developed various models and measures to assess mental wellbeing, differing in the degree to which they emphasize hedonic or eudaimonic foundations.

Diener defines subjective wellbeing (SWB) as comprising two components: affective and cognitive evaluations of one’s life (Diener et al., 2009). The affective component aligns with the hedonic perspective by experiencing positive or negative emotions (Diener, 1984). To assess this dimension, the frequently employed Positive and Negative Affect Schedule conceptualizes positive and negative emotions as distinct and independent constructs (Watson et al., 1988), demonstrating strong validity also for older adults (Kercher, 1992). The cognitive evaluation, on the other hand, represents an evaluative assessment of one’s life, often measured using the Satisfaction with Life Scale, that aims to capture a person’s overall life satisfaction (Diener et al., 1985).

In contrast to Diener’s hedonically inspired model of subjective wellbeing (Diener, 1984), Ryff’s model of psychological wellbeing (Ryff and Keyes, 1995) offers an alternative perspective. She argues that an affect-based approach, which emphasizes happiness and life satisfaction, fails to capture psychological functioning and overall mental wellbeing. Her model is inspired by eudaimonic principles and comprises six dimensions: *Autonomy, Environmental Mastery, Personal Growth, Positive Relations with Others, Purpose in Life*, and *Self-Acceptance*. These are assessed using Ryff’s Scales of Psychological Wellbeing (Ryff and Keyes, 1995).

While these models highlight different facets of wellbeing, with Diener focusing on hedonic and Ryff on eudaimonic aspects, Seligman (Seligman, 2011) proposes a more integrative approach. Unlike the dichotomy of hedonia and eudaimonia, he combines elements from both perspectives, offering a comprehensive understanding of human flourishing by defining five building blocks of wellbeing: *Positive Emotion, Engagement, Relationships, Meaning*, and *Accomplishment* (PERMA). *Positive Emotion* aligns with the hedonic perspective, emphasizing pleasure and life satisfaction, while the remaining components are rooted in eudaimonic principles, focusing on personal growth, purpose, and fulfillment. By integrating these dimensions, PERMA presents a holistic view of psychological wellbeing, acknowledging that both immediate happiness and long-term psychological fulfillment is essential for overall flourishing. Importantly, Seligman (Seligman, 2011) emphasizes that no single element defines wellbeing on its own. Rather, each contributes independently and is often pursued for its own sake. Based on this, the PERMA-Profiler assesses each of the five building blocks with three items (Butler and Kern, 2016). This scale has demonstrated effectiveness in assessing the wellbeing of elderly in an Iranian sample (Payoun et al., 2020) and is validated in German (Wammerl et al., 2019).

Having outlined three established models of wellbeing, we now discuss which is most appropriate for assessing the impact of social robots on older adults. While enhancing long-term wellbeing remains the ultimate design objective, measuring short-term effects is equally important, as situational wellbeing offers valuable insights for improving robotic applications.

Given that emotions play a central role in human-robot interaction (Spitale and Gunes, 2022), Ryff’s (Ryff and Keyes, 1995) eudaimonic model may overlooks this crucial aspect. Furthermore, dimensions such as *Personal Growth*, *Purpose in Life*, and *Self-Acceptance* are difficult to influence through short-term robotic interventions, making it challenging to evaluate application effects without longitudinal studies. Therefore, the other two models, SWB and PERMA, appear better suited for evaluating the effects of social robots on older adults, as both emphasize emotional experience.

Interestingly, Goodman et al. (Goodman et al., 2018) found that SWB and PERMA capture essentially the same underlying construct of wellbeing. Thus, either framework could validly serve as a basis for assessing the impact of social robot applications on senior wellbeing. However, compared to SWB, PERMA offers a more nuanced approach, enabling detailed analyses of wellbeing across different dimensions. This granularity makes PERMA particularly advantageous for evaluating situational wellbeing during human-robot interactions.

For instance, researchers measuring the impact of social robots may not observe immediate changes in overall life satisfaction, since it is cognitively evaluated over a longer period. However, they may detect variations in momentary wellbeing during specific interventions. PERMA allows comparisons between a person’s overall wellbeing and their situational wellbeing. For example, when playing a game with a robot and reaching a higher level, a person may experience a sense of *Accomplishment*. Researchers can best capture this momentary contribution to well-being by measuring the five building blocks of wellbeing. This approach helps them understand how different applications may address individual elements of wellbeing to varying degrees. Moreover, this approach provides insights on where applications have potential for optimization. To illustrate, if an application scores high on engagement but low on positive emotions, integrating touch from the robot could enhance the emotional experience (Sawabe et al., 2022).

Further support for applying the PERMA framework in the context of social robots can be found in the reviews by Kachouie et al. (Kachouie et al., 2014; Kachouie et al., 2017), who categorized a range of wellbeing outcomes, such as reduced loneliness and depression, within the dimensions of PERMA. More recently, a PERMA-based wellbeing intervention for college students significantly improved emotion regulation and had a positive impact on mood (Laban et al., 2025). These findings highlight the potential of PERMA as a valuable foundation for designing social robot applications aimed at promoting wellbeing.

## 3 Conceptual model

**FIGURE 1:**
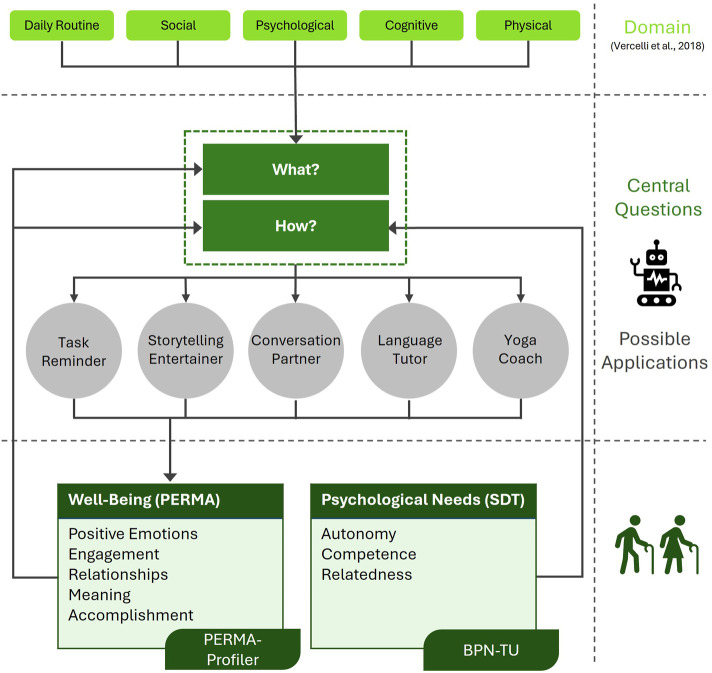
Conceptual model for designing social robot applications in senior care, integrating Self-Determination Theory and the PERMA model.

Having established the theoretical foundation, we now introduce our conceptual model. Our goal is to support designers and researchers in developing robotic applications for older adults by proposing which types of applications are particularly relevant and how they should be designed. The model is structured around two central guiding questions: *What?* and *How?* Before elaborating on the model in detail, we provide a graphical overview in Figure 1.

### 3.1 What to design?

Current companion robots tend to focus on specific domains (Zhao et al., 2023). To address the question of *What?* social robots should support, researchers should consider the domain in which their application is rooted to meaningfully benefit older adults. Therefore, we aim to identify key areas where social robots can be effectively integrated into senior care. Bemelmans et al. (Bemelmans et al., 2011) highlighted the potential of social robots at the social, psychological, and physiological levels. While these areas are highly relevant, we argue that additional domains, such as cognitive training (Lim, 2023) or medication reminders (Prakash et al., 2013) require separate consideration. Therefore, we base our conceptual model on the five domains identified by Vercelli et al. (Vercelli et al., 2018) in their comprehensive review: *Daily Activities, Social, Psychological, Cognitive*, and *Physical*. We slightly adapt the first category, renaming it to *Daily Routine* to better reflect the supportive role of social robots in task structuring and reminders, rather than in the direct execution of tasks. In Table 1, we provide examples of existing work and offer an outlook on potential future applications.

**TABLE 1:** Examples of existing work and possible future applications in selected domains.

Domain	Examples of existing work	Possible future applications
Daily Routine	Robots help with reminders, for medication (Gasteiger et al., 2022; Huang and Huang, 2021) or appointments (Bartl et al., 2016)	A nudging application that reminds to drink enough water on a hot day; a reminder application to water plants in the garden
Social	Scheduled encounters with the robot Pepper increased the degree of social interactions between care home residents (Blindheim et al., 2023); active engagement with Paro can provide social support (McGlynn et al., 2017)	A storytelling application that encourages to share the story with others; a relatedness application that motivates to ask fellow home care residents for personal information
Psychological	Older adults regard companionship as an important feature of social robots such as Kompaï (Zsiga et al., 2013)	A conversation application for talking and providing companionship; a meditation application to reduce stress or anxiety
Cognitive	Robots offering games are appreciated for cognitive stimulation (Gasteiger et al., 2021b; Banck et al., 2025)	A learning application for acquiring a new language; a quiz application including different fields of knowledge
Physical	Encouraging movement by mirroring the robot Nao is positively received (Preston et al., 2024)	A yoga application for improving strength and balance; a bedtime application for improved sleep onset

The PERMA dimensions inform both the *What?* and the *How?* in our model. We begin by focusing on how PERMA guides the *What?*. While new applications can be derived by examining key domains for robotic intervention as explained above, they may also emerge from the building blocks of wellbeing themselves. For example, a researcher might concentrate on the wellbeing dimension *Accomplishment*, aiming to create an intervention that fosters a sense of achievement. This could lead to a robot that proposes daily challenges, such as exercising on Monday, journaling on Tuesday, and calling one’s grandchildren on Wednesday. By completing these tasks, seniors may experience an enhanced sense of *Accomplishment*. The resulting application then spans multiple domains, as its basis for idea generation lies in a specific wellbeing dimension rather than a domain-driven starting point.

### 3.2 How to design?

Having addressed how PERMA can guide the generation of application ideas, we now turn to the *How?*, by illustrating how the dimensions of wellbeing can serve as guiding principles for application design. For instance, a reminder application within the domain of *Daily Routine* may encourage older adults to regularly open their windows for fresh air. Rather than issuing a directive, the application can be designed in line with the PERMA-dimension *Meaning* by framing the action as an act of caregiving. The robot might say, *‘I need some fresh air - could you please open the window for me?’*. By responding to this request, older adults are not merely completing a task but may experience a heightened sense of meaning through caring for the robot.

Additionally, SDT also informs the *How?* aspect of application design, particularly by emphasizing the fulfillment of the three basic psychological needs: autonomy, competence, and relatedness. These needs serve as key design principles, as their satisfaction contributes to wellbeing. Autonomy can best be supported by offering choices. For example, in a storytelling application, the robot could enhance autonomy by enabling older adults to influence the progression of the narrative through interactive decision-making. Competence may be fostered by integrating small riddles into the story, accompanied by positive reinforcement for correct answers and supportive hints when needed. Relatedness can be addressed by having the robot use the listener’s name and encouraging them to share the storytelling experience with friends or family.

### 3.3 Measuring the effects

To ensure that applications developed using our conceptual model can be tested in the field, standardized and consistent measures for evaluating the interactions are essential. These allow for the comparison of effects across different application types and domains. We therefore propose the use of two validated instruments to assess both wellbeing and the satisfaction of basic psychological needs.

General wellbeing should be measured before the interaction using the PERMA-Profiler (Butler and Kern, 2016). To assess the impact of the interaction itself, we suggest adapting the PERMA-Profiler to capture situational wellbeing. For example, the item “In general, how often do you feel joyful?” can be modified to “How often did you feel joyful during the game with the robot?”. This enables researchers to directly compare baseline and situational wellbeing and identify which dimensions of wellbeing are effectively supported. In cases of low outcomes in specific dimensions, the design can be refined to better target these building blocks of wellbeing. If interactions consistently score high across all PERMA dimensions, on-going interaction with the robot could help maintain or even improve long-term wellbeing in seniors.

In addition to assessing wellbeing, it is also important to evaluate how effectively different applications support the basic psychological needs defined by SDT. While some applications may naturally support specific needs more than others, a consistent evaluation across all three is desirable. For this purpose, we recommend the Basic Psychological Needs Scale for Technology Use (BPN-TU), available in both German and English. This instrument has been specifically developed to measure need satisfaction in human-technology interactions and has been validated involving the social robot Pepper as a rehabilitation tool. Notably, the BPN-TU not only captures relatedness to other people but also to the robot itself (Moradbakhti et al., 2024), making it especially suitable for evaluating social robot applications.

Together, the (adapted) PERMA-Profiler and the BPN-TU provide standardized, comparable metrics across diverse interventions. They support systematic field testing and enable researchers to assess the effectiveness of different applications while identifying areas for improvement.

## 4 Future work

Given the growing shortage of care workers, the development of meaningful social robot interventions for seniors is becoming increasingly vital. In our future work, we aim to iteratively design at least one application per domain, guided by the principles of our proposed conceptual model. This includes integrating both the PERMA framework and Self-Determination Theory into the design process and evaluating each application using the corresponding validated measures. By doing so, we also intend to test the model’s practical applicability and enhance the comparability of different social robot interventions, ideally extending to applications developed by others. Eventually, our goal is to further establish the model as a standard for designing and evaluating social robot applications in senior care that explicitly aim to promote wellbeing.

## Data Availability

The original contributions presented in the study are included in the article/supplementary material, further inquiries can be directed to the corresponding author.
